# Protein Supplementation to Augment the Effects of High Intensity Resistance Training in Untrained Middle-Aged Males: The Randomized Controlled PUSH Trial

**DOI:** 10.1155/2017/3619398

**Published:** 2017-06-01

**Authors:** Andreas Wittke, Simon von Stengel, Michael Hettchen, Michael Fröhlich, Jürgen Giessing, Michael Lell, Michael Scharf, Michael Bebenek, Matthias Kohl, Wolfgang Kemmler

**Affiliations:** ^1^Institute of Medical Physics, Friedrich-Alexander University of Erlangen-Nürnberg, Henkestraße 91, 91052 Erlangen, Germany; ^2^Department of Sports Science, University of Kaiserslautern, Erwin Schrödinger-Straße, 67663 Kaiserslautern, Germany; ^3^Institute of Sports Science, University of Koblenz-Landau, Fortstraße 7, 76829 Landau, Germany; ^4^Department of Radiology and Nuclear Medicine, Nürnberg Hospital, PMU, Prof.-Ernst-Nathan-Straße 1, 90419 Nürnberg, Germany; ^5^Department of Radiology, University Hospital of Erlangen, Maximiliansplatz 3, 91054 Erlangen, Germany; ^6^Department of Medical and Life Sciences, University of Furtwangen, Jakob-Kienzle-Straße 17, 78054 Villingen-Schwenningen, Germany

## Abstract

High intensity (resistance exercise) training (HIT) defined as a “single set resistance exercise to muscular failure” is an efficient exercise method that allows people with low time budgets to realize an adequate training stimulus. Although there is an ongoing discussion, recent meta-analysis suggests the significant superiority of multiple set (MST) methods for body composition and strength parameters. The aim of this study is to determine whether additional protein supplementation may increase the effect of a HIT-protocol on body composition and strength to an equal MST-level. One hundred and twenty untrained males 30–50 years old were randomly allocated to three groups: (a) HIT, (b) HIT and protein supplementation (HIT&P), and (c) waiting-control (CG) and (after cross-over) high volume/high-intensity-training (HVHIT). HIT was defined as “single set to failure protocol” while HVHIT consistently applied two equal sets. Protein supplementation provided an overall intake of 1.5–1.7 g/kg/d/body mass. Primary study endpoint was lean body mass (LBM). LBM significantly improved in all exercise groups (*p* ≤ 0.043); however only HIT&P and HVHIT differ significantly from control (*p* ≤ 0.002). HIT diverges significantly from HIT&P (*p* = 0.017) and nonsignificantly from HVHIT (*p* = 0.059), while no differences were observed for HIT&P versus HVHIT (*p* = 0.691). In conclusion, moderate to high protein supplementation significantly increases the effects of a HIT-protocol on LBM in middle-aged untrained males.

## 1. Introduction

Since most sedentary people give time constraints as the main hindrance to frequently exercise [1], time-effective exercise protocols may increase people's willingness to complete exercise doses necessary to affect health and fitness related outcomes [2]. Low volume, high intensity endurance, and resistance exercise protocols may be time-efficient methods to realize this aim [3–5]. Indeed, with respect to resistance exercise, most authors reported moderate positive effects on muscle mass [6] and strength [7, 8] after single set training protocols. Although there is an ongoing discussion [8–10] with respect to the relative effectiveness of single set protocols, based on recent meta-analysis [6–8, 11], the more time consuming multiple set protocols (MST) were reported to generate (significantly) higher exercise effects on body composition and strength development compared with single set protocol [6–8, 11]. This can be specifically applied when protocols only differ for a number of sets [12], a condition that ensures a proper comparison of both methods.

Protein supplements generally augment the adaptive response of muscle mass and strength to resistance-type exercise [13–16]. Consequently, additional protein supply should augment the effect of single set, or single set based HIT, resistance exercise on muscle mass and strength to a higher (potentially multiple set) level. Based on the evidence-based expectation [6–8, 11] of the general superiority of MST versus single set protocols even when both approaches were applied to muscular failure, the aim of the study was to determine whether additional protein supplementation increases the effect of HIT on muscle mass, strength, and body fat to an equal MST-level.

Hence, our primary hypothesis was that protein supplementation significantly increases the effect of HIT on muscle mass to an MST-level. The secondary hypothesis was that protein supplementation significantly increases the effect of HIT on muscle strength to an MST-level. An experimental hypothesis was that protein supplementation did not significantly affect body fat changes between the exercise groups.

## 2. Methods

### 2.1. Trial Design

The Physical adaptions in Untrained on Strength and Heart (PUSH) study was a 22-week randomized controlled exercise trial with a 2 × 2 parallel group design using an incomplete cross-over approach. PUSH focused on the effect of high intensity, single set resistance exercise protocols (HIT) with and without protein supplementation versus high intensity, multiple set resistance exercise protocols (HVHIT) versus sedentary control on muscle and strength parameters in untrained males 30–50 years old. The Institute of Medical Physics (IMP), Friedrich-Alexander University Erlangen-Nürnberg (FAU), Germany, initiated the study that was conducted from April 2012 to July 2013. The study was approved by the ethics committee of the FAU (Ethikantrag number 53_12 B) and the Federal Bureau of Radiation Protection (Z5-22462/2-2012-060). PUSH complied with the Declaration of Helsinki “Ethical Principles for Medical Research Involving Human Subjects.” After detailed information, all participants gave written informed consent. The study was registered under https://www.clinicaltrials.gov (NCT01766791).

### 2.2. Outcomes

#### 2.2.1. Primary Study Outcome

- Bone-free lean body mass (LBM) changes as determined by Dual Energy X-Ray Absorptiometry (DEXA) from baseline to the end of the intervention after 22 weeks of exercise.

#### 2.2.2. Secondary Study Outcome

- Appendicular muscle mass (ASMM) changes as determined by DEXA from baseline to the end of the intervention after 22 weeks of exercise.
- Dynamic leg and hip extensor strength changes as determined by an isokinetic leg press from baseline to the end of the intervention after 22 weeks of exercise.

#### 2.2.3. Experimental Study Outcome

- Total body fat (%) changes as determined by DEXA from baseline to the end of the intervention after 22 weeks of exercise.

### 2.3. Changes of Trial Outcomes after Trial Commencement

Originally, the primary study endpoint was “fat- and bone-free, cross-sectional area of the mid thigh.” However, due to problems with the segmentation software and due to the amount of time passed since the study end we consider “bone-free lean body mass” (LBM) as an alternative primary study endpoint of this contribution.

### 2.4. Participants

**Figure 1:**
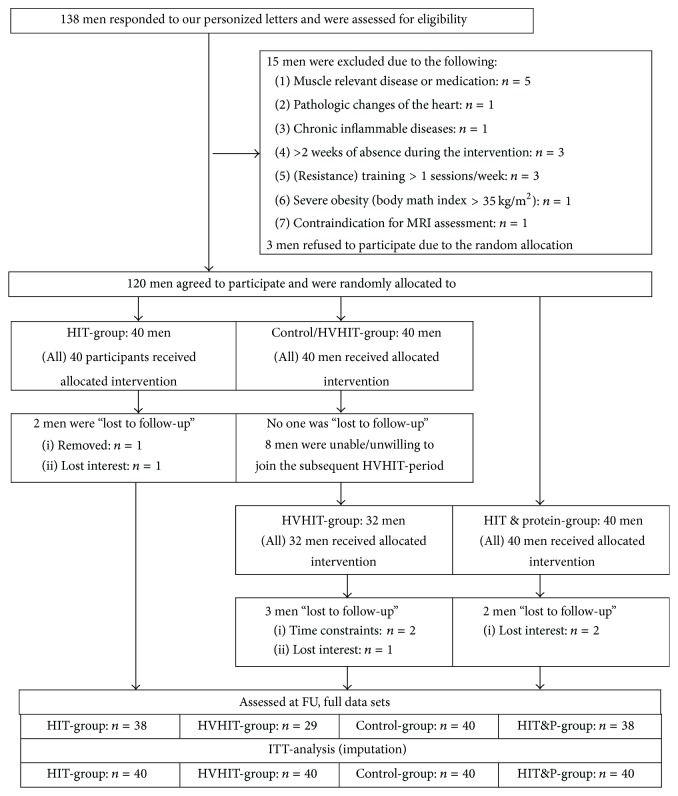
Flow-chart of the PUSH study.

Participant flow of the study is given in Figure 1. Two-thousand randomly selected men between 30 and 50 years living in the area of Erlangen, Germany, were contacted using the citizen's register of the municipality. In personalized letters, we gave detailed study information including the most relevant eligibility criteria (e.g., training status, Body Mass Index (BMI), and absence during the interventional period of the study). From the 138 men who responded and were further assessed by the principle investigators for eligibility, 15 subjects had to be excluded. Reasons for exclusion were (a) “trained status” (i.e., >1 resistance exercise session/week; ≥2 total exercise sessions/week during the last 2 years; *n* = 3), (b) pathological changes of the heart and acute inflammatory diseases (*n* = 2), (c) diseases or medication affecting muscle metabolism (e.g., acute glucocorticoid therapy; *n* = 5), (d) severe obesity (BMI-cut point > 35 kg/m^2^; *n* = 1), (e) more than 2 weeks of absence during the interventional phase (*n* = 3), and (f) contraindication for Magnetic Resonance Imaging (MRI) assessment (*n* = 1). However, of the 123 men remaining, 3 were unwilling to be randomly allocated to a group and quit the study. Thus, 120 men were randomly allocated (see Figure 1) to the three study groups.

### 2.5. Sample Size

As given, the sample size calculation of the PUSH study was originally based on another primary study endpoint. With respect to the present study endpoint “bone-free lean body mass” the sample size of 40 participants per group enables us to detect a difference of (ES) 0.11 ± 0.15 with 90% power (*α* = .05) (*t*-test based sample size calculation). This difference was adapted from Krieger [6] for single set versus 2-3 set approaches using the highest variance (SD) reported in his analysis.

### 2.6. Randomization Procedures

**Figure 2:**
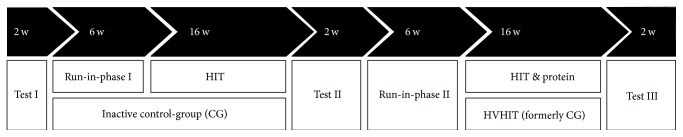
Study design of the PUSH study.

Stratified for age (4 stratas of 5 years), 120 participants were randomly assigned to three study arms (a) high intensity, low volume (resistance exercise) training (HIT) group; (b) HIT and protein supplementation; and (c) waiting-control-group/high intensity/high volume (resistance exercise) training (HVHIT) (Figures 1 and 2; Table 2) by a uniform allocation rate (1 : 1 : 1) (Figure 1). For the allocation, lots were drawn by the participants themselves. Each of the 120 lots was put in opaque plastic shells (“kinder egg,” Ferrero, Italy) and drawn from a bowl. Neither participants nor researchers knew the allocation beforehand. Finally, group status of the participants was listed and participants were assigned to the different study arms by the principle investigators.

### 2.7. Blinding

Outcome assessors and research assistants were blinded, that is, not informed with respect to the group status of the participant, and were not allowed to ask.

### 2.8. Intervention

The study design is presented in Figure 2. The first study section focused on the comparison of HIT with the control-group (CG) which was requested to maintain their lifestyle, physical activity, and exercise habits during this phase. After 22 weeks of intervention, a short testing period, and a break of 1 week, the former CG crossed over and performed the high volume/high intensity resistance exercise training (HIHVHIT), while the former HIT-group was not further monitored. Additionally, a HIT and protein (HIT&P) group was set up and exercised in parallel to the HVHIT-group for a total of 22 weeks (Figure 2). Of importance, the 22-week control assessment of the CG was also the baseline test for the HIT&P-group.

As intended, HIT and HVHIT varied solely in the number of sets (HIT: 1 set versus HVHIT: 2 sets). Thus, the resistance exercise strategy of the present study will be described en bloc below.

The exercise training was carried out in two well-equipped commercial gyms with identical resistance exercise devices. We offered mandatory core hours (7:00–9:00; 12:00–14:00; 17:00–21:00) of exercise training 7 days a week during which participants had to exercise. Certified instructors and/or research assistants, all of them carefully briefed by the principal investigators, consistently supervised all the sessions and checked the proper application of the exercise protocol including the aspect of “work to failure.” We aimed to realize an instructor to participant ratio of 1 : 5; however due to unexpected variations this ratio varied between 1 : 1 and 1 : 8.  Table 1 gives an overview of the resistance exercise protocol applied in this project.

#### 2.8.1. Resistance Exercise Protocol

During the initial phase, all the resistance protocols started with two weeks of familiarization and briefing and a further four weeks of conditioning. During the first two sessions of the familiarization period, we carefully explained and trained the proper execution of the exercises, applying an instructor: participant ratio of 1 : 2. After this 2 × 90 min introduction, we focused on the participant's ability to choose an “adequate load” for the prescribed range of repetitions (Table 1).

During the conditioning phase we applied consistently two sessions/week, with 10 varying exercises, 1-2 sets of 10–15 repetitions (reps), a time under tension of 2 s concentric, 1 s isometric and 2 s eccentric (2 s-1 s-2 s), and an incomplete work to failure (maximum effort minus 2-3 reps; recently defined as non-Repetition Maximum (nRM) [17]). Certified instructors consistently supervised all sessions and checked the proper application of the exercise protocol including the aspect of “work to failure” (Table 1).

After the conditioning phase, we applied a linearly periodized 16-week resistance exercise program with four 4-week phases with each 4th week as a recovery week. The exercise programs generally consisted of two to (rarely) three consistently supervised sessions/week. Thus, the average training frequency for the intervention period was 2.14 sessions/week. All the main muscle groups were addressed by 10–13 exercises/session taken from a pool of 17 exercises (latissimus back and front pulleys, front chin ups, seated rowing, back extension, inverse fly, hyperextension, sitting bench press, shoulder-press, military press, butterfly with extended arms, crunches, leg press, leg extension, leg curls, leg adduction, and abduction) conducted over the full range of motion on resistance devices (MedX, Ocala, FL, USA). The number of repetitions varied between 3 and 12 reps. Intensity of the exercise was prescribed as a range of repetitions (e.g., 6–8 reps) that had to be accomplished under the premise of work to momentary muscular failure (MMF; [17]); this was however progressively intensified by the advanced techniques (MMF+) listed below. Time under tension was similarly manipulated in all exercise groups within a range between explosive concentric movements, 1 s isometric and 3 sec eccentric and 4 s-1 s-4 s. Time under tension was negatively correlated with number of reps; that is, 3-4 reps were conducted with very slow movement velocity (4 s-1 s-4 s). We conducted a standardized warm-up that focused on the muscle group that was subsequently addressed. Thus, a warm-up set on a chest press constituted warming up for pectorals, triceps, and deltoids. Six to eight repetitions with ≈50% 1RM using a 3-1-3 time under tension (TUT) were performed only once per muscle group (not per exercise); thus, also only one warm-up set was performed per synergistic block (Table 1).

Applying the above described protocol, phase 2 (weeks 7–10) focused on work to momentary muscular failure (MMF [17]) with rest periods of 2-3 min between exercises/sets. However, after week 8 we reduced the rest periods to 1 min between exercises, in order to further increase the general intensity of the protocol.

During phase 3 (weeks 11–14) we added a superset strategy with one session/week prescribing a synergistic approach (4 blocks of 2–4 exercises for the same muscle group performed consecutively) while the second (or third) sessions/week focused on an antagonistic approach (5 blocks of one exercise each for agonist and antagonist performed consecutively). Rest periods between the synergistic and antagonistic exercises were ≤1 min while rest periods between the blocks were 2 min (Table 1).

During phase 4 (weeks 15–18) we also enhanced the set-end point and muscular effort by prescribing further (forced) reps with reduced weight (−10–15%) immediately (<20 s) after the initial set to MMF (“drop sets”). Rest periods between all exercises either within or between the blocks were 2 min. Finally, during phase 5 (weeks 19–22), each second session, the load was reduced twice (e.g., −10% work to MMF and again −10% work to MMF). Rest periods were ≤1 min within the synergistic/antagonistic blocks and 90 s- 2 min between the blocks. Movement velocity during phases 4 and 5 was consistently prescribed at (TUT) 3 s-1 s-3 s (Table 1).

#### 2.8.2. HIT versus HVHIT Resistance Protocol

As mentioned above, we prescribed HIT as a “single set to failure protocol with advanced techniques.” By definition, “single set” refers to the exercise not to the muscle groups; thus the same muscle group may be addressed by several exercises each performed once. In the HVHIT-group we applied the identical exercise protocol, however consistently with two sets per exercise; thus the volume of the HVHIT was exactly twice as high compared with the HIT-protocol. Of importance, we organized HVHIT as a circuit, that is, the second set/exercise was not conducted immediately after the first set but after the first bout of all the exercises.

Thus, in summary the number of sets (HIT: 1 set/exercise versus HVHIT: 2 sets/exercise) was the only difference between the two resistance exercise protocols.

We provided training logs for all the training phases that prescribed the training session in detail. Besides proper completion of the logs, for example, with respect to number of reps and corresponding load achieved, participants were asked to list the net exercise time and to list their rate of perceived exertion (Borg CR 10 Scale) [18] for the corresponding session. In order to properly determine training attendance, we used chip cards that gave subjects entrance and exit to the gym and thus monitored their gross stay.

### 2.9. Protein Supplementation

We aimed to realize a protein supply of 1.5–1.7 g/kg body weight per day in the HIT&P-group. Based on the 4-day dietary protocol described below, participants with a protein intake <1.5 g/kg/d (*n* = 32) were provided with protein supplements (43 ± 19 g/d). The protein powder used in this study (protein4you, Saarlouis, Germany) consisted of multicomponent protein (whey, casein, egg, and soya) with a chemical score of 156. One hundred (100) g contained 76.5 g of protein, 3.9 g of carbohydrates, and 3.2 g of fat resulting in a calorific value of 363 kcal/100 g protein powder. One portion of 30 g was enriched with 500 mg L-carnitine and 3 g of L-leucine. Participants of the HIT&P-group were requested to ingest the prescribed dose accurately on a daily base and to split doses higher than 30 g/d. Compliance with prescribed protein powder intake was regularly queried during the exercise sessions.

### 2.10. Testing

We conducted all tests in a blinded fashion. Baseline and follow-up assessment of the participant were conducted at the same time of the day (±1 h).

Height (Holtain, Crymych Dyfed., Great Britain) and body weight were measured using calibrated devices (InBody 230, Seoul, Korea). Body composition was assessed by Dual Energy X-Ray Absorptiometry (DEXA, QDR 4500a, Discovery-upgrade; Hologic Inc., Bedford, USA) using standard protocols [19]. Appendicular skeletal muscle mass (ASMM) was calculated using the bone-free lean mass of the upper and lower limbs as segmented by the standard segmentation protocol of the manufacturer. Based on the daily “phantom” assessments and automatically calculated by the software, long-term reliability of our DEXA device was 0.77% for the intervention period.

Maximum isokinetic strength of the leg and hip extensors was tested using a ConTrex isokinetic leg press (Physiomed, Laipersdorf, Germany). Bilateral leg and hip extension was performed in a sitting, slightly supine position (15°), supported by hip and chest straps. Range of motion was selected between 30° and 90° of the knee angle, with the ankle flexed 90° and positioned on a flexible sliding footplate. The standard default setting of 0.5 m/s was used. After familiarization with the movement pattern and standardized warm-up (10 reps with ≈50% 1RM with a 2 min break after the warm-up) participants were asked to conduct five repetitions with maximum voluntary effort. Participants conducted two trials intermitted by two minutes of rest. We consistently included the higher value of both trials in the data analysis. Applying this approach, reliability for the maximum leg press test (test-retest-reliability; intraclass correlation) in this cohort was 0.88 (95%-CI: 0.82–0.93).

Briefly, one repetition maximum (1RM) was calculated using a repetition to fatigue (RTF) predicting equation specifically designed for this cohort. During the second week of the conditioning period, four pairs of 1RMs and corresponding RTFs were performed for leg press, bench press, rowing, and latissimus pulleys. 1RM-tests were conducted according to the test protocol applied by Kraemer et al. [20]. To determine RTFs at different intensities, participants were asked to select loads that permitted repetitions in the range of 2–4, 5–7, and 8–11 using a 2-1-2 s TUT. For the prediction of the 1RM, a cubic regression polynomial was computed, taking into account all test values. For a more detailed description of our proceeding the reader is referred to another recent article, albeit covering a different cohort [21].

Demographic parameters, health risk factors, and physical activity were sampled by validated baseline questionnaires [22, 23]. In order to determine changes of medication, diseases, lifestyle habits, physical activity, exercise, dietary pattern, and nutritional supplementation, (i.e., parameters that may affect the study endpoints), the same questionnaires were used at follow-up.

The participants' dietary intake was assessed immediately before and after the trial by 4-day dietary protocols conducted by all participants. The consumed food was analyzed using the Freiburger Ernährungs-Protokoll [Freiburger Nutrition Protocol] (nutri-science, Hausach, Germany). In case of dubious results (i.e., energy consumption <1000 or >3500 kcal; *n* = 6) participants were correspondingly interviewed, briefed again, and asked to properly complete another dietary protocol based on more representative days.

### 2.11. Statistical Analysis

An intention to treat (ITT) analysis included all the participants who were randomly assigned independently of compliance or lost to follow-up. R statistics software [24] was used in combination with multiple imputation by Amelia II [25]. The full data set was used for multiple imputation, with imputation being repeated 100 times. Overimputation diagnostic plots provided by Amelia II confirmed that the multiple imputation worked well in all cases. Based on a statistically and graphically checked normal distribution of the primary and secondary outcomes presented here, dependent *t*-tests were used to analyze within-group changes. One-way ANOVA was applied to determine differences between the groups, where we used the approach of Alison [26] to combine the results of the imputed data sets. In case of relevant differences, pairwise *t*-test comparisons with pooled standard deviation were conducted [27]. The *p* values obtained in the pairwise comparisons were adjusted for multiple testing by the method of Holm [28], hence keeping the family-wise error rate under control. All tests were 2-tailed; significance was accepted at *p* < 0.05 or adjusted *p* < 0.05, respectively. Effect sizes were calculated using Cohen's *d*.

## 3. Results

Table 2 gives the baseline characteristics of the participants. Of importance, in line with this ITT-approach that included all participants in the analysis, the CG-group follow-up data were taken as the HVHIT-group baseline data (Figures 1 and 2), which explains the differences in baseline characteristics between these groups.

No significant differences (*p* > 0.30) were determined between the groups.

Of importance, eight participants of the CG were either unable (*n* = 2) or unwilling (*n* = 6) to cross-over to the HVHIT-group (Figure 2); thus 32 men started exercising in the HVHIT. A further two participants of the HIT and HIT&P each and three participants of the HVHIT were lost to follow-up while all of the CG-group participated in the FU assessment (Figure 1). The reasons for withdrawal were (a) relocation (*n* = 2), (b) time constraints due to paternity or occupational changes (*n* = 3), and (c) loss of interest (*n* = 2). Attendance rates among the exercise groups were relatively high and averaged around 95 ± 5%. Average rate of perceived exertion (RPE) for the sessions (phases 2–5) was comparable between the groups (6.8 ± 1.0, Borg CR 10; 7 = very hard). In detail, RPE increased significantly (*p* = 0.004) from 6.3 ± 0.7–6.5 ± 0.6 in phase 2 to 7.0 ± 0.6–7.2 ± 0.6 in phase 5 with no group differences for any phase. Average net duration of the exercise training (phases 2–5) differed significantly (*p* = 0.001) between HIT/HIT&P (36.6 ± 2.4 min) versus HVHIT (74.7 ± 3.1 min). No injuries occurred during the exercise sessions.

Thirty-five (92%) participants of the HIT&P-group said they had taken the protein supplements exactly according to our prescription and two participants slightly more (≈10–20%) and one participant slightly less (≈−20%) protein than specified. Thus, based on the protein supply and the dietary protocol, total protein intake of the HIT&P-group averaged 1.64 ± 0.10 g/kg/d at study start and 1.61 ± 0.12 at study end. Only one participant did not reach the specified protein intake of at least 1.5 g/kg/d (1.44 g/kg/d). Apart from regular muscle soreness, no negative side effects of resistance exercise and/or protein supplementation were reported.

At baseline, no significant differences were determined for the primary and secondary study endpoints. Bone-free lean mass (LBM, Table 3) increased significantly in all exercise groups (*p* ≤ 0.043); however only HIT&P and HVHIT differ significantly from control (*p* ≤ 0.002; *d*′ = 0.98–1.02). Further, HIT diverges significantly from HIT&P (*p* = 0.017; *d*′ = 0.67) and varied nonsignificantly from HVHIT (*p* = 0.059; *d*′ = 0.60), while no differences were observed for HIT&P versus HVHIT (*p* = 0.69; *d*′ = 0.10). Comparable results were determined for ASMM (Table 3), a region of interest where changes of bone-free-LBM can be completely related to changes of muscle mass. Thus, although we determined only a nonsignificant difference (see above) between HIT and HVHIT, we confirmed our primary hypothesis that protein supplementation significantly increases the effect of HIT on muscle mass.

Table 4 gives the results for the secondary and experimental study endpoint. Dynamic knee extensor strength significantly (*p* < 0.001; *d*′ = 0.84–1.02) increased in all exercise groups and was maintained in the CG. Further, all exercise groups significantly differ from control (*p* ≤ 0.01), while no significant differences for leg and hip extensor strength changes were observed between the exercise groups. Thus, we have to reject our hypothesis that protein supplementation significantly increases the effect of HIT on muscle strength (to an MST-level).

Total body fat decreased (HIT, HVHIT: *p* ≤ 0.002 and HIT&P: *p* = 0.058) in the exercise groups and was maintained in the CG. However, only the HIT-group differed significantly from control (*p* = 0.002; *d*′ = 0.85), while no significant differences with respect to changes of body fat rate were observed between the exercise groups. Thus, we confirmed our secondary hypothesis (a) that body fat rate was similarly reduced in all exercise groups compared with the nontraining control-group.

No changes of diseases, medication, or general physical activity were reported during the intervention period. Contrary to the commitment given, two subjects of the CG as well as two of the HIT-group and one participant of the HVHIT engaged in endurance exercise programs (30–90 min/week). Also against the protocol, three subjects of the HIT and one subject each of the HVHIT-group and HIT&P reduced their energy intake by 10–20%. In summary, energy consumption of the HIT-group decreased significantly (*p* = 0.001) by 66 ± 120 kcal/d (−2.5 ± 4.1%), while energy intake remained stable in the HVHIT (0.2 ± 6.1%), HIT&P (0.9 ± 4.4%), and CG (1.7 ± 5.5%). In parallel, dietary protein consumption decreased slightly in the HIT (−2.2 ± 11.4%, *p* = 0.192) and increased in the HIT&P (1.9 ± 13.3%; *p* = 0.574), HVHIT (1.6 ± 13.7%; *p* = 0.925), and CG (3.7 ± 8.8%. *p* = 0.016). Between-group differences were observed for energy intake (HIT versus CG; *p* = 0.027). Using a per protocol analysis and excluding the participants who infringed the study protocol with respect to lowering energy intake (HIT: *n* = 5, HIT&P: *n* = 1, HVHIT: *n* = 2, and CG: *n* = 2) did not affect the results on primary or secondary endpoints. Even after excluding all the HIT-participants with an energy intake reduction of >105 kcal/d (which is the upper limit of the 95% CI of the energy intake change of the HIT-group), there were no differences in our results. A more individualized analysis also demonstrated that all the subjects who reduced energy intake by more than 5% consistently ranged in the upper third of baseline energy intake (i.e., >34.0 kcal/kg bodyweight/d).

## 4. Discussion

The primary study aim of this contribution was very pragmatic: will additional protein supplements enhance the effect of HIT to the level equal to the more time consuming, but also more effective, multiple set resistance exercise protocols? Addressing the scientific basis for our approach that multiple set protocols were more favorable to increase muscle mass, we marginally failed to determine significant differences of HIT versus HVHIT for hypertrophy parameters (i.e., LBM: *p* = 0.059), at least in the main analysis. This result can largely be attributed to the adjustment for multiple testing; a direct comparison of both groups determined a significant superiority of HVHIT (*p* = 0.018). However, it was not our goal to compare identical single and multiple set resistance protocols prescribed to MMF+ [17], but to enhance the results of the time-efficient HIT by a low-threshold dietary intervention. The primary finding of this study that additional protein supplementation significantly augments the hypertrophic response of a HIT resistance exercise protocol generally confirmed our hypothesis, although some limitations, discussed below, may decrease the value of this observation. Reviewing the literature, two recent meta-analyses [13, 14] focus on the issue whether additional protein supplementation increases muscle mass parameters more than exercise alone. Cermak et al. [13] determined a mean difference of 0.75 kg LBM in 6–24 weeks (95% CI: .42–1.10 kg) for combined protein supplements and young untrained subjects. Naclerio et al. [14] who focus on whey protein reported an overall increase around 1.3 kg in 6–12 weeks (95% CI: −0.6–3.3 kg). Of importance, none of the groups included in the meta-analysis consumed less than ≈1.2 g/kg bodyweight at baseline [29]. The most prominent difference between resistance exercise protocols with and without protein supplementation in untrained males (19 ± 2 years) was reported by Willoughby et al. [30]. The authors determined a 2.8 kg difference after a multiple set, high intensity (3 sets 6–8 reps at ≥85% 1RM) resistance protocol applied 4 times/week (splitting protocol) for 10 weeks with (40 g/d) or without (40 g/d dextrose) supplementation with whey protein. For resistance-trained younger subjects (24 ± 7 years) Burke et al. [31] and Cribb et al. [32] reported significant effects of whey protein (≈100–110 g/d) and exercise versus exercise alone (1.4 kg and 1.6 kg after 6 or 11 weeks, resp.); however, the effect was much more pronounced when creatine monohydrate was also added (3.1 kg, *p* < 0.05 [31], and 2.6 kg [32]). Coming closest to our protocol, however, Mielke et al. [33] compared the effects of a single set plus protein (26.2 g whey protein including 6.2 g leucine) versus a 2-set protocol (6–8 reps with 80% 1RM) without protein for 8 weeks in untrained young males 19–28 years and did not observe significant differences between the training groups. However, due to the low sample size, the lack of an isolated HIT-group, a suboptimum body composition assessment, a short study duration, and an unintended reduction in energy (120–550 kcal/d) and dietary protein (10–23 g/d or 10–22%) this study is hard to interpret.

In contrast to our results (Table 4), increases of 1RM for leg press given by the meta-analysis of Cermak et al. [13] cited above differed on average by ≈13.5 kg or 33% (95% CI: 6–21 kg; 6–24 weeks) and were up to 2 times higher [30] with versus without additional protein supplementation. Further, most studies reported higher strength gains after multiple sets versus single set protocols [7], at least when comparing otherwise identical exercise protocols. However, the corresponding effect seemed to be more pronounced in athletes and after long-term training periods [8].

Independently of protein supplementation and exercise volume, all the exercise groups lost body fat (*p* = 0.058–0.001). Albeit not significantly different between the exercise groups, the effect was most prominent in the HIT-group (−1.2 kg). Initially, we attributed this result to the significant reduction in energy intake (−2.5 ± 4.1%) in this group; however, several subanalyses that excluded and compared subjects who increased their energy expenditure and/or decreased energy intake during the intervention did not result in a relevantly different data constellation for body fat changes or other endpoints. Thus, although it would seem obvious that the significant changes of energy intake of the HIT-group would explain their prominent body fat reductions, statistical evidence rejected this simple association.

Reviewing the relevance of protein supplement in this context, although not consistent across the present studies, slightly more prominent fat reduction was reported by protein supplements added to resistance exercise [13].

In order to allow the scientific community to estimate the relevance, evidence, and generalizability of our results, some particular features and limitations of the study will be addressed.

(1) With respect to the study design, a consequent parallel group design would have been the most appropriate choice for addressing our research issue. However, due to limited resources and the high sample size required (*n* = 40) we decided to divide the study into two study periods. In the first study period, we focused on the general effectiveness of a HIT-protocol, while in the second we focused on the main topic of the project, the comparison of the HIT&P versus the HVHIT-group. Although from a biometrical point of view this procedure is a viable one, caution is warranted during a comprehensive comparison of all groups.

(2) Unlike most studies which supplied carbohydrates (CHO) as a placebo, we did* not* apply this approach for ensuring “isocaloric conditions.” While protein and amino acids are essential for anabolic processes, unlike CHO, their significance for energy metabolism during energy balance in healthy adults is minor [34]. Further, considering protein-induced thermogenesis, satiety, and decreased energy-efficiency [35], supplying the same dose of protein versus CHO as applied by most studies (i.e., [30–33, 36]) may generate a significant bias.

(3) The overall protein intake of ≈1.6 ± 0.1 g/kg (HIT&P) was in the upper range of recent recommendations for athletes [37] and exceeded the dietary intake of the other study groups (HIT, HVHIT) by 30–40%.

(4) Drawing lots may be considered to be not the most sophisticated randomization strategy. However, our recent studies determined that drawing lots and thus randomizing “oneself” boosted acceptance of a nonfavored study group, a very important aspect in nonblindable intervention studies [38, 39].

(5) The slight but significant reduction in caloric intake of the HIT-group can be considered as the main limitation of this study. Although several statistical approaches did not indicate that this caloric reduction was a relevant confounder of the primary and secondary outcome, an uncertainty remained as to whether the results of the HIT and corresponding group differences can be fully explained by the exercise intervention.

(6) Further, the high number of CG participants (20%) who were either unable or unwilling to cross-over to the subsequent HVHIT-protocol (Figure 2) may have confounded our results. However, since we did not communicate the subsequent resistance protocol to the CG until the end of study phase 1, the unwillingness to cross-over (*n* = 6) can be considered as an indication of the aversion to conduct the time consuming HVHIT-protocol.

(7) With respect to generalizability, we took a homogeneous cohort of untrained middle-aged males for whom the relevance of time-efficient exercise protocols may be of particular interest. With respect to other, less motivated and able-bodied cohorts, the exhaustive MMF(+) approach might aggravate the application of HIT, even though similar protocols have been successfully applied in older people.

## 5. Conclusion

Due to the complexity of the study project and limited space, we were unable to discuss all the important aspects of the project in adequate depth here. However, the central aim of this study was an advanced “proof of concept,” without the intention of addressing potentially underlying physiologic mechanisms in depth. In summary, time-efficient HIT resistance exercise protocols combined with moderately dosed protein supplementation may be a good choice for motivated people with low time budgets who want to improve their body composition, strength, and cardiometabolic health [40].

## Figures and Tables

**Table 1:** Exercise protocol. TUT: time under tension; exs: exercises; reps: repetitions; RegW: regeneration week; SuS: superset; expl: explosive (very fast) movement velocity.

Time	Number of reps (break)	Work to failure strategy	TUT (s)
Phase 1			
Weeks 1-2	Familiarization phase	Incomplete work to failure	2-1-2
Weeks 3–6	Conditioning phase: 10 exs 1-2 sets 10–15 reps (90 s)	Incomplete work to failure	2-1-2

Phase 2	8–10 reps (break 2-3 min)	MMF	expl-1-2
Weeks 7–10	5–7 reps (break 2 min)	MMF	2-1-2
	3–5 reps (break 60 s)	MMF	4-1-2
	RegW: 10–12 reps (break 2 min)	MMF -3 reps	2-1-2

Phase 3	See phase 2, however, break	See phase 2 + supersets	expl-1-2
Weeks 11–14	between SuS-exercises: 60 s; between SuS	(1) Session/week agonists SuS^1^	to
	“blocks” 2 min	(2) Session/w. antagonists SuS^2^	4-1-4
	Week 4: RegW (see phase 2)	2–4 exercises per SuS “block”	2-1-2

Phase 4	See phase 3	See phase 3 and drop-set with a single load reduction of −10–15% for each exercise immediately after work to MMF	3-1-3
Weeks 13–18	2 min breaks		
	Week 4: RegW		

Phase 5	See	See phase 3 and drop-sets with a twofold reduction of −10 and −10% for each exercise immediately after work to MMF and MMF+	3-1-3
Weeks 19–22	phase 3		
	Week 4: RegW		

**Table 2:** Baseline characteristics of the PUSH study groups.

Variables	HIT (*n* = 40)	HIT&P (*n* = 40)	HVHIT (*n* = 40)	Control (*n* = 40)	*p*/*F* value
Age [y]	42.9 ± 5.4	43.7 ± 5.9	42.9 ± 5.6	42.5 ± 5.6	0.717/.451
Body Mass Index	27.5 ± 4.0	27.3 ± 4.1	26.9 ± 4.2	26.8 ± 4.3	0.376/.770
Total body fat mass [kg]	25.6 ± 5.1	25.0 ± 4.8	24.9 ± 6.1	24.9 ± 6.0	0.904/.172
Physical activity [index]^1^	2.9 ± 1.4	2.8 ± 1.3	2.7 ± 1.2	2.7 ± 1.1	0.818/.311
Exercise volume [min/week]	28.4 ± 35.9	40.1 ± 41.8	35.0 ± 37.9	35.0 ± 38.2	0.640/.564
Working time [h/week]	43.4 ± 3.2	44.5 ± 2.9	43.5 ± 3.1	44.1 ± 3.5	0.322/1.17
Upper/middle/lower class [%]	20/68/12	25/63/12	18/73/9	18/73/9	≥0.816/.94^2^
University studies [%]	70	78	75	75	0.785/1.07^2^
Smokers [*n*]	1	1	2	2	0.733/3.59^2^
Diseases (ICD-10) [%]	20	25	22	21	0.963/.29^2^
Chronic medication [%]	10	13	13	13	0.981/.18^2^
Energy intake [kcal/day]^3^	2658 ± 723	2703 ± 662	2559 ± 592	2516 ± 758	0.671/.518
Protein intake [g/kg/d]^3^	1.13 ± 0.29	1.21 ± 0.39	1.17 ± 0.40	1.12 ± 0.34	0.294/1.25
Fat/CHO/alcohol [g/d]^3^	99/305/12	106/314/12	100/286/14	94/298/14	≥0.20/.199

^1^Based on a scale from 1 (very low) to 7 (very high) according to a subjective assessment of professional, household, and recreational activities. ^2^Chi square test value; ^3^based on a 4-day dietary intake protocol. CHO: carbohydrates.

**Table 3:** Baseline values and changes of LBM in the study groups. ^*∗*^*p* < 0.05; ^*∗∗∗*^*p* < 0.001; n.s.: nonsignificant.

LBM [kg]	HIT MV (95%-CI)	HIT&P MV (95% CI)	HVHIT MV (95% CI)	CG MV (95% CI)	*p*
Baseline	67.2 (64.8–69.6)	67.8 (65.1–70.4)	65.7 (63.2–68.2)	65.6 (64.1–68.1)	0.53
Difference	0.45 (.15–.85)^*∗*^	1.38 (.95–1.81)^*∗∗∗*^	1.24 (.76–1.73)^*∗∗∗*^	0.04 (−.38–.45)^n.s.^	0.001

**Table 4:** Baseline values and changes of ASMM, isokinetic leg and hip extensor strength, and total body fat-rate in the study groups.^*∗*^*p* < 0.05; ^*∗∗*^*p* < 0.01; ^*∗∗∗*^*p* < 0.001; n.s.: nonsignificant.

	HIT MV (95%-CI)	HIT&P MV (95% CI)	HVHIT MV (95% CI)	CG MV (95% CI)	*p*
*Appendicular skeletal muscle mass (ASMM) [kg/g]*					
Baseline	31.24 (30.0–32.4)	31.22 (29.9–32.6)	30.19 (28.9–31.5)	30.17 (29.0–31.4)	0.44
Difference	200 (−29–429)^n.s.^	797 (557–1037)^*∗∗∗*^	581 (299–862)^*∗∗∗*^	15 (−219–249)^n.s.^	0.001

*Isokinetic leg and hip extensor strength [N]*					
Baseline	3239 (3027–3481)	3323 (3145–3501)	3315 (3153–3519)	3288 (3101–3475)	0.61
Difference	446 (253–637)^*∗∗∗*^	418 (279–555)^*∗∗∗*^	334 (239–429)^*∗∗∗*^	27 (−56–111)^n.s.^	0.001

*Total body fat [%]*					
Baseline	25.61 (24.0–27.3)	25.00 (23.5–26.6)	24.97 (22.7–26.9)	24.9 (23.1–27.1)	0.51
Difference	−1.20 (−.75–1.67)^*∗∗∗*^	−.44 (.05–.90)^n.s.^	−.86 (−.31–1.40)^*∗∗*^	0.06 (.50–.39)^n.s.^	0.001
